# The political economy of the African pharmaceutical sector's “industrial underdevelopment” lock-in: The importance of understanding the impact of persistent colonial extractive institutions

**DOI:** 10.3389/frma.2023.1020588

**Published:** 2023-02-09

**Authors:** Geoffrey Banda

**Affiliations:** ^1^The Innogen Institute, Science Technology and Innovation Studies, University of Edinburgh, Edinburgh, United Kingdom; ^2^Tacking Infections to Benefit Africa (TIBA), University of Edinburgh, Edinburgh, United Kingdom; ^3^Innovation for Cancer Care in Africa (ICCA), University of Edinburgh, Edinburgh, United Kingdom

**Keywords:** extractive institutions, political economy, African industry development, African pharmaceutical sector, innovation systems

## Abstract

Improved African pharmaceutical manufacturing has been on global and local agendas since the 1970s, yet the industry has been locked-in into low technologies for decades. What caused the technological and industrial stagnation for such a critical sector for local and global health security? What are the political economy roots of such long-running industrial underdevelopment lock-in? What do colonial extractive economic and political institutions and their setup and mixes have to do with the sector? This study considers how extractive economic and political institutions' architectures and infrastructures shaped the African pharmaceutical industry's underdevelopment. We argue that extractive economic and political institutions shaped contemporary institutions in former colonial countries, and these institutions persist for a long time. The pivotal argument of innovation systems is that technological change-driven innovation is important for building superior economic performance and competitiveness, and institutions are a vital component of the system. However, institutions are not value-neutral; they carry the political and economic objectives and aspirations of the agents who design them. Innovation systems theory needs to incorporate the analysis of extractive economic and political institutions and the role they played in locking-in the African pharmaceutical industries into underdevelopment.

## 1. Introduction

African pharmaceutical manufacturing is not a new phenomenon; early vaccine manufacture setups date back to the 1890s, and drug manufacturing establishment through chiefly multinational subsidiary/affiliate companies started in the early 20th century. Indigenous-owned drug manufacturing companies took off after independence as multinationals left. However, the footprint is still small (Banda et al., 2022), despite improved local manufacturing being on global and local agendas since the 1970s (Turshen, 2001). To date, there have been no significant advances in the production of active pharmaceutical ingredients (API) for drugs, active drug substances for vaccines, advanced drugs and vaccines, and medical devices, and international competitiveness. COVID-19 unraveled the sector's low technological capabilities and pandemic unpreparedness. International supply chains for medicines, medical devices, and diagnostics were disrupted causing acute shortages of raw materials, price escalations, and relegation of African countries to the back of COVID-19 vaccine queues (Banda et al., 2021a,b).

What events shaped these technological capabilities and industrial development path dependences that led to underdevelopment for a sector that is so critical for assuring local and global health security? What are the colonial political economy roots of such long-running industrial underdevelopment lock-in? What do colonial extractive economic and political institutions and their setup and mixes have to do with the sector's problems? Acemoglu and Robinson (2013) argue that inequality in the world is largely a consequence of uneven adoption and dissemination of technologies and that inclusive economic and political institutions foster sustainable economic activity, productivity, and economic growth. This is achieved through Schumpeterian creative destruction, which is driven by innovation arising from technological change. We argue that because colonial African countries did not have inclusive political and economic institutions (Acemoglu and Robinson, 2013), the economy, institutions, and industrial structures worked for the benefit of the empire. They were designed to support the empire's industrial production systems and trade objectives. Governance of these global industrial production systems (value chains) and division of labor entrenched particular technological effort path dependences, which, through institutional persistence even after independence, locked-in industrial underdevelopment.

The bedrock of innovation systems theory is that technological change-driven innovation offers a better explanation for superior economic performance and international competitiveness. Consequently, there is great emphasis on public policy, institutional arrangements, and science, technology, and innovation (STI) institutions' role in stimulating and sustaining technological change (Lundvall and Archibugi, 2002; Lundvall, 2007), which drives Schumpeterian creative destruction, which is essential for sustainable economic growth (Acemoglu and Robinson, 2013). However, innovation systems theory is silent on colonial extractive political and economic institutions' contexts and their impact on innovation institutions and infrastructures in Africa. The theory is silent on how empire and colonial political and economic objectives shaped innovation and industrial infrastructures and subsequently situated and locked them in at particular subservient global value chain positions, leading to persistent industrial underdevelopment. Arocena and Sutz (2000, 2002), while focusing on Latin America, give us the earliest glimpse of the application of the national innovation system (NIS) to developing country contexts and their insights on interdisciplinary analysis of contemporary underdevelopment challenges. They focused on the role of social actors and processes of interaction and contexts driven by political, institutional, cultural, and economic factors. They argue that NIS, as an ex-post concept, carries normative weight, embodies relational aspects, is a policy subject and describes situations where conflict exists. We argue that incorporating political economy and global value chain analytical approaches may offer a better understanding of the challenges of applying NIS to the African pharmaceutical sector lock-in into underdevelopment because persistent extractive economic and political institutions shaped historical and contemporary industrial structures' path dependences.

The political economy focuses on who determines who has power and resources and how they are distributed in society, thereby determining political and economic outcomes. Adler (2011) defines political economy as “the combined and interacting effects of economic and political structures or processes…” and further asserts, “capitalistic economic structures and market forces influenced and were influenced by political power at local, national and international levels”. This is useful in unraveling how colonial global value chains, the international division of labor, and industrial activities circumscribed and locked-in periphery industrial structures into lower value chain positions because of deep dependences between economic and political phenomena (Adler, 2011). Ake (1983) asserts that industrial capitalism tended to lead to imperialism, and underdevelopment theory, dependence theory, or center-periphery theory all belong to political economy approaches. The political economy thus encompasses analysis of actors, institutions, structures, power, and behavior that is shaped by incentives, values, belief systems, ideas, and value-laden interests (Andreas et al., 2021). Of the four political economy approaches, namely, Austrian; Institutionalist; Contemporary or Modern Marxist School; and the Modern Utilitarian or Public Choice School (Gandy, 1992), we use the institutionalist approach with some elements of contemporary approaches. We take this approach because economic behavior, economic organizations, and economic and political policies do not operate in a vacuum but are shaped by and, in turn, shape social contexts (Boettke et al., 2013).

This study sets out to consider how extractive economic and political institutions' architectures and infrastructures shaped the African pharmaceutical industry's underdevelopment. The main argument is, because of institutional persistence, what colonial extractive institutions were created for matters. Extractive economic institutions extract wealth or incomes from one section of society to benefit a narrow subset of society. However, inclusive economic institutions generate positive feedback through two engines of prosperity: technology and education. Inclusive institutions are “sufficiently centralized and pluralistic”. There is a strong synergy between inclusive political and inclusive economic institutions (Acemoglu and Robinson, 2013, p. 76).

We focus on four areas: first, the scarcity policy design capabilities for the pharmaceutical sector at the national level; second, financing innovation and research, industry development, and entrepreneurship institutional challenges; third, the importance of building new institutions that support and de-risk innovation early stages development; and fourth, building strong local regulatory capabilities for emerging technologies. Our main argument is that current manifestations of industrial underdevelopment and path dependencies are consequences of progenitor colonial extractive institutions that have persisted to date. The application of innovation systems theory to African countries should account for these idiosyncratic dynamics.

The rest of the paper is set out as follows: Section 2 provides a brief background, section 3 covers literature review and section 4 presents the methodology. Section 5 presents the results followed by discussion in section 6. Chapter 7 concludes the paper.

## 2. Background

The political economy of the African pharmaceutical sector is inextricably linked to the roots of empire industrial production systems, labor division, asymmetric trade patterns, and hegemonic power wielded in colonial global value chain governance. The underlying colonial institutional dynamics persist perversely into contemporary times. Innovation systems theory ignores these complex institutional dynamics immanent in current industrial underdevelopment path dependencies. Protagonists would argue that we should look forward and forget the past. This is problematic because institutional history matters. Looking forward without dismantling the persistent roots of colonial extractive institutions is problematic because foreign colonial legacy institutions called foreign introduced exogenous institutions (FEX) by Boettke et al. (2008) possess low institutional stickiness for radical institutional upgrading and renewal. History also matters because institutions are carriers of history (David, 1994) and idiosyncratic traditions that shaped particular path dependences and evolution of particular rules of the game. History matters from three perspectives: first, history shapes mutually consistent expectations; second, there are highly durable assets that require information channels and codes [institutions] to operate efficiently in organizations; and third, complex human organizations have interrelated constituencies that interact in complex ways to constrain or avail choices based on particular rules or procedures [institutions] that usually rely on compatibility and consistency [path dependence] (David, 1994).

### 2.1. Colonial institutions were designed to serve settler or extractive empire goals

Economic institutions, political institutions, and incentives are important determinants of wealth or poverty (Acemoglu and Robinson, 2013). Acemoglu et al. (2001) proposed a theory of institutional differences amongst countries colonized by Europe based on three premises because different colonization policies created different types of institutions. Where they encountered extractive systems, they established themselves as the new elite and took over taxation, tribute, and forced labor (Acemoglu et al., 2001). The Spanish colonization of Mexico and Latin America was based on resource extraction and labor coercion (Acemoglu and Robinson, 2013, p. 10). The purpose of these states was the transfer of resources to the metropole, for the benefit of an individual [King Leopold in Belgium], the state [Spain and Portugal], and the private enterprises [Britain]. These resource transfer institutions set up a chain reaction of detrimental investment and economic development trajectories (Acemoglu and Robinson, 2013, p. 10).

The Spanish model did not work in North America because the indigenous communities could not be exploited. Consequently, the colonists had to work themselves. This ultimately led to the realization that the colonists needed incentives such as property rights and a say in political systems for things to work. Economic and political participation led to economic growth. In the USA, this led to a constitution that espoused democratic principles, created limitations on the use of political power, and distributed that power broadly in society (Acemoglu and Robinson, 2013, p. 28).

In developing countries, European colonialism created institutional reversals (Acemoglu et al., 2002). Colonization strategies were shaped by the feasibility of settlement. High disease incidence and fatality areas did not favor settlement and the converse is true. In high-disease areas, extractive institutions arose, and in those colonies with extractive institutions, they were extended. This had a huge impact on the types of industries that arose from these different institutional setups (Acemoglu et al., 2001, 2002). These institutions have persisted to date, and there is a “strong correlation” between these early institutions and current institutions. The thesis of institutional persistence (Acemoglu et al., 2001) dates back to work on how “control structures” setup by the Ottoman, Russian, and Chinese empires persisted for over 500 years, as evidenced in the work of Wittfogel (1957) and others, such as La Porta et al. (1998) and Coatsworth (1999). This underscores the need to consider the political economy of extractive institutions in innovation systems theory application to African countries.

Extractive institutions can be “equilibrium institutions”; however, they deter sustainable industrial and economic development (Acemoglu et al., 2002). Consequently, decolonisation of problematic former colonial institutions is important because powerful colonial and post-independent political elites shaped and perpetuated extractive economic and political institutions that do not foster Schumpeterian creative destruction but serve a few. They were designed to optimize the externalization of resources and wealth. Contemporary agents may knowingly or ignorantly perpetuate “underdevelopment” approaches.

### 2.2. Financial institutions and their critical role in economic development

How financial institutions influence industry development and modernization, trade, and commerce has been explored in development literature for a while (Foxwell, 1917; Cameron, 1967; Cohen, 1967). Patrick (1966) and King and Levine (1993) demonstrate the close link between a country's financial system and economic growth. In the early development stages, bank-based financial systems support economic growth at a faster rate compared to market-based systems (Levine, 2002). The lack of financial systems deepening has been a critical institutional barrier to industrial development. Banks are important catalysts for industrialization (Da Rin and Hellman, 2002) because they promote the creation of new industries; however, to do this, the banks should be of sufficiently large size, with sufficient market power and the ability to mobilize huge resources. Alternative catalysts of industry development are governments and corporations. Thus, banks play a pivotal function in supporting the development of new markets, industries, and economic growth. Bank day-to-day processes of mobilizing savings, evaluating projects, managing risk, monitoring managers, and facilitating transactions are important drivers of technological innovation and economic development (Da Rin and Hellman, 2002). However, access to finance does not solve all the issues. Finance capability, knowing who to borrow from, what type of borrowing instrument, and the ability to negotiate terms and conditions are critical especially in the African pharmaceutical sector (Banda, 2013a).

Financial history literature shows that financial systems deepening causes economic development and vice versa. Financial systems deepening (development of financial systems and increased financial services provision) promotes economic growth (supply leading concept), and conversely, economic development could accelerate financial systems development (demand following concept) (King and Levine, 1993). This dual causality contributes more to the causal relationships in developing countries than in industrialized countries, where financial deepening accelerates economic growth based on rapid capital accumulation and productivity growth (Calderon and Liu, 2002). Developing country contexts tend to have the supply-leading phenomenon (Patrick, 1966). This is important for our argument because extractive economic and political institutions did not create deep financial systems architectures. Lack of financial deepening results in high-interest charges and spreads, equity finance scarcity, and specialization in short-term lending and not long-term lending, which is critical for capital expenditure (capex) funding (Fafchamps et al., 1995; Beck and Hesse, 2009; Beck et al., 2009, 2011; Andrianova et al., 2010, 2011a,b; Fowowe, 2011). Capex funding scarcity is the Achilles heel that fuels pharmaceutical industrial underdevelopment on the continent.

## 3. Literature review

### 3.1. National innovation systems

Technological change-driven innovation is a key driver of international economic competitiveness and specialization. That is why innovation and interactive learning offer better explanations for why some companies, sectors, regions, and nations do better than others (Lundvall, 2007). Edquist (1997, p. 14) defined innovation systems as “all important economic, social, political, organizational, institutional and other factors that influence the development, diffusion and use of innovations”. Other definitions emphasize that NIS is composed of a network of institutions [public or private] that interact to drive the development, adoption, modification, and diffusion of new technologies and ways of doing things (Freeman, 1987; Nelson, 1993). The notion of NIS was developed at the same time in the USA, by Nelson, and in Europe, by Freeman, who worked with the IKE group in Aalborg in the early 1980s (Lundvall, 2007). Lundvall (1992) emphasized the role of interaction and learning by doing, focusing on the nation-state. He used three major building blocks: sources of innovation [learning and search and exploration]; types of innovation [radical and incremental]; and non-market institutions [user producer interactions and institutions]. Technological change is best understood as an outcome of NIS (Lundvall, 1992; Nelson, 1993), of which institutions are a key component.

(Schrempf et al., 2013, p. 6–7) argue that institutions are important because they serve as frameworks for implementing policy, are targets for policy intervention, and have instrumentality. Instrumentality emanates from three perspectives: they shape the types of innovation choices and rate of technology take-off; they have directionality; and they embody the values and aspirations of agents [economic and political actors]. David (1994) adds the fourth dimension. Institutions are carriers of history and they shape the form and function of path dependence because “the present is coupled up with history” in the routines, conventions, and traditions of economic activities and technological efforts. Therefore, if institutions persist for a long time, are transmitted across colonial and post-independence political and economic regimes, and they are structurally problematic, then subsequent efforts to modify such institutions that ignore history will fail.

Nelson (1993) focused on how institutions work by understanding how and why particular setups of actors collaborate. He looked at institutions in STI and how they interact and assist tertiary institutions as knowledge and technology generators. This is particularly pertinent to our argument because colonial setups were characterized by a lack of collaboration between industry and universities. MacLeod (1996) points out that British and Indian science suffered from poor contact with manufacturing interests. STI institutions on the continent were set up primarily for agriculture, mining, and health based on resource extraction economic policies. This institutional historical path dependence is omitted in innovation systems theory. The omission is understandable. Contemporary innovation systems theory arose from studying Japan post WWII (Arocena and Sutz, 2000) and then spread to other western countries and developing countries (Watkins et al., 2015). Its application to African contexts needs in-depth engagement with colonial institutional legacies and their persistence.

Innovation systems literature points to determinants of technological change being embedded in societal structures, and that societal aspirations and objectives through institutions shape technological change. It is not surprising that institutions feature prominently in sectoral and technological innovation system innovations. In sectoral systems of innovation, institutions shape the interaction of agents that produce a set of new or established products for specific uses, utilizing specific knowledge bases, technologies, and inputs, as well as demands (Malerba, 2002). Three dimensions are important: knowledge and technological domain; actors and networks; and institutions. Institutions include the norms, rules of the game, how the game is played, and standards amongst others. Emphasizing the importance of institutions to technological innovation systems (TIS), Carlsson and Stankiewicz (1991) define TIS as “a dynamic network of agents interacting in a specific economic/industrial area under a particular institutional infrastructure [our emphasis] and involved in the generation, diffusion, and utilization of technology”. Thus, institutions and their infrastructures are essential for innovation generation and the production of new technologies. Lundvall and Archibugi (2001) further elaborated on the TIS definition to include the role of “a set of institutions and regulations characterizing the rules of behaviors and knowledge infrastructures connected to it” to stimulating the creation of new technologies. Institutions are, however, not value-neutral. We argue that colonial institutional infrastructures were never set up to develop internationally competitive and innovative African pharmaceutical industrial sectors.

### 3.2. Foreign exogenous introduced institutions were extractive

Institutional path dependence is shaped by culture and values which influence new institutions' adoption success (Boettke et al., 2008). Boettke et al. (2008) present the notion of institutional stickiness. It is based on a taxonomy of three institutions' categories: Indigenous Introduced Endogenous (IEN) Institutions, Indigenous Introduced Exogenous (IEX) Institutions, and Foreign Introduced Exogenous (FEX) Institutions. IEN institutions are designed by locals, which usually arise spontaneously, and embody local “norms, customs and practices that have evolved informally over time in specific places”. IEX institutions, on the other hand, are associated with internal policies designed by, for example, the national government, and FEX institutions, on the other hand, are those introduced by foreigners to a local place, for example, policies developed by the development community. Exogenous institutions are characterized by their development outside a certain place and subsequent imposition. They use examples of policies created by, for example, IMF, USAID, or the World Bank as exogenous institutions. Institutional stickiness and, therefore, the likelihood of success of any proposed institutional change is a function of the new institution's position in relation to indigenous agents of prior eras. In their explanation of how and why history matters for institutions, Boettke et al.'s (2008) conceptual framework focuses on the ability or inability of new institutional arrangements to take root when transplanted on old ones.

We find this notion attractive for this paper because colonialism was based on the political economy of FEX institutions which (Acemoglu et al., 2001, p. 220) termed as extractive institutions that were imposed on colonies by empire. That these extractive FEX institutions became equilibrium institutions and persisted to contemporary times is undeniable. Their original economic, global value chain, and trade objectives locked colonies into industrial underdevelopment.

Ake (1983) quoting Marx asserts that imperialism led to colonization and the resultant settlements served to grow raw materials for the industrialising metropole in the late 19th and 20th century. He further argues that from a political economy perspective “imperialism has been and remains a decisive influence on the nature and the possibilities of the periphery” (Ake, 1983, p. 32). The varieties of capitalisms and institutions used during colonial eras cannot be divorced from their global phenomenon and resultant asymmetric power in trade and economic relations between the metropole and periphery. This institutionalized international division of labor has not changed much. Innovation system theory needs to critically engage with these issues and offer to unlearn and dismantle innovation and technology conceptual frameworks informed by colonial economic development doctrines.

### 3.3. Colonial doctrines of STI, industrial, and economic developments still haunt the present

Colonial science had instrumentality and it served the empire (MacLeod, 1996). It emphasized agriculture and biology (botany, zoology, and ecology), tropical medicine, and anthropology knowledge systems. These fields were important for settlement and supporting FEX STI institutions that promoted local agricultural and mining industries for raw material exports. These institutions persist and manifest in the current funding dynamics for STI on the continent, which favor agriculture and [global] health (see, for example, Chataway et al., 2019). Decades later, well after independence, the effects of the FEX extractive institutions are reflected in the economic structures of many African countries which heavily depend on the export of raw materials (agricultural and mining) to international markets with little local beneficiation or value addition. This is not a coincidence, but a product of persistent colonial extractive institutional infrastructures and economic policies. Worboys and Petitjean (1996) shed some light on the long-term historical links of this phenomenon. They argued that the empire had three spheres: domains composed of Canada, USA, New Zealand, and Australia; India, which then included Pakistan, Bangladesh, and Burma; and the Colonial Empire made up of Crown Colonies and Protectorates. The latter two spheres had FEX extractive institutions imposed on them. The provision of raw materials and agricultural products to be traded for manufacturers from Britain essentially forced these countries into a state of “underdevelopment” by circumscribing and locking-in their industrial activities to upstream value chains (the commodities sectors) with the least economic value and low international competitiveness.

The three spheres of empire served to advance inter-imperial trade and political relations, all driven by the doctrine of complementary development. Inter-imperial trade and political relations—clear examples of political economy in terms of power, agency, and resource allocation—had objectives of solidifying the empire. Around 1918, during “constructive imperialism,” huge capital investment in transport and infrastructure through loans and grants was availed to facilitate trade and develop the agricultural sector in the colonies (Worboys and Petitjean, 1996). Thus, the thrust of colonial development policy was to integrate colonial economies into dependant relationships with Britain, as argued earlier, thereby locking them into a state of industrial underdevelopment. What is particularly illuminating for our arguments is the focus on science training. Disciplines that favored industrial development in that era such as chemistry, engineering, and physics were actively funded and supported in the metropole. Whereas, for the colonies, disciplines that fostered easier settlement, biology and tropical medicine to reduce disease fatalities, and agriculture and mining to improve raw material extraction were encouraged (Worboys and Petitjean, 1996). Thus, the early STI institutions and structures were set up to meet three objectives: discovering agricultural and mining economic opportunities; making the tropical environments safe to live in for colonialists; and solving technical problems in low-value production systems for local agricultural, food, and mining industries to fulfill exports of raw materials to industrial sectors at the center.

Hodge (2011) argued that the post-war development drive reflected imperial policy to boost local productivity and output. The imperial government drove the commodity value chains that encouraged higher local productivity for food and raw materials to meet British and colonial needs. Of particular importance to this study, MacLeod (1996) observed that British and Indian science suffered from poor contact with manufacturing interests. The same phenomenon can be claimed for Africa, more so in the pharmaceutical sector (see local API manufacturing challenges section).

Kothari (2006) asserts that there is pervasive continuity of these science policy and development ideologies. Colonial discourses, cultures, and practices continued and pervaded post-independence development thinking and doing of development. Kothari (2006) bases her argument on the continuity of expertise and ideologies through the vehicle of colonial officers, who during post-colonial and independence eras had to re-engineer themselves and sought new careers in development and academia. They became consultants to the emerging development industry and trained incoming administrators in academic institutions. Kothari then argues that there is an inherent continuation of the “doctrines of [colonial] development and ancestry of ideas” which still haunts the present. Kothari (2006) quoted Said's (1994) statement, “The past is rarely over and done with but haunts the present”. From the era of colonial development approaches to development cooperation and the emerging international development approaches, the actors and agents, as well as the agenda setters have remained the same, and so have the institutions of economic and industrial development as well as those for STI.

### 3.4. Global value chains and their governance

Understanding the roots of industrial underdevelopment lock-in through global value chain governance helps unravel their transmission mechanisms. Kaplinsky and Morris (2002) define a value chain as “the full range of activities that is required to bring a product or service from conception, through the different phases of production, delivery to final customers, and final disposal after use”. Most of the pharmaceutical companies on the continent were set up as subsidiaries of MNCs. Gereffi et al. (2005) show that in such situations, the hierarchy global value chain governance model type is the most prevalent for multinationals characterized by vertical integration. The dominant form of governance is achieved through managerial control with asymmetric power flow from managers at headquarters to subordinates at the subsidiary or affiliate. It is not difficult to understand the transmission mechanism of industrial underdevelopment lock-in in that case. The headquarters' international division of labor placed subsidiaries at particular positions on the global value chains. For contemporary local manufacturers, the captive value chain governance approach is dominant, although others could argue that the market governance approach could be applied. They are small buyers who are dependent on large suppliers of APIs and excipients, who are vertically integrated. Consequently, they cannot compete on price or scale on local and international tenders. They find it hard to vertically integrate because the local institutional frameworks' support for new technologies is fragmented (see findings section).

## 4. Methodology

This paper draws on empirical evidence from three studies: doctoral work during 2010–2012 on financing ARV (Antiretroviral) drugs manufactured in Zimbabwe; an ESRC project REGenableMed that focused on the UK's Regenerative Medicine sector's business models, value chains, and innovation ecosystems which ran from 2014 to 2017; and an ESRC project—Innovation in Cancer Care (ICCA) which ran from 2019 to 2021. In addition, it draws from numerous conversational interviews and discussions at workshops and conferences on African pharmaceutical production. The work is based on primary data and secondary literature from the history of science and colonial economic institutional setups. The study is based on the case study approach with empirical data collected from industrialists, policy-makers, and experts from the NGO and health-related sectors using semi-structured interviews. In addition, this study draws from conversational interviews with policy and pharmaceutical executives at workshops and conferences in Ethiopia, Kenya, Tanzania, South Africa, the UK, Zimbabwe, Morocco, and South Africa. This study joins up various strands of empirical work to understand the current “what” is happening and why questions in the sector, and draws from secondary sources to explore explanations of why historical extractive institutional architectures could have led to the current state of African pharmaceutical industrial stagnation and underdevelopment.

## 5. Findings

### 5.1. Institutions for pharmaceutical policy design are scarce at the national level

Supranational organizations have been dominant promoters of local pharmaceutical production policies and strategies. They include the African Union (AU), African Union Development Agency/New Partnership for Africa's Development (AUDA/NEPAD), Southern Africa Development Community (SADC), and regional economic groupings such as the East African Community (EAC) amongst others. International development organizations such as the United Nations Industrial Development Organization (UNIDO), the German Development agency GIZ, and WHO, for example, have also been active. The AU's Pharmaceutical Manufacturing Plan of Action (PMPA) was supported by UNIDO, and so were Nigeria, Kenya, Zimbabwe, Ghana, and Uganda's local manufacturing country surveys. In addition, UNIDO supported the production of six strategy documents that spanned vaccine and drug production, cGMP roadmaps, and pharmaceutical manufacturing plans of action.

**Figure 1:**
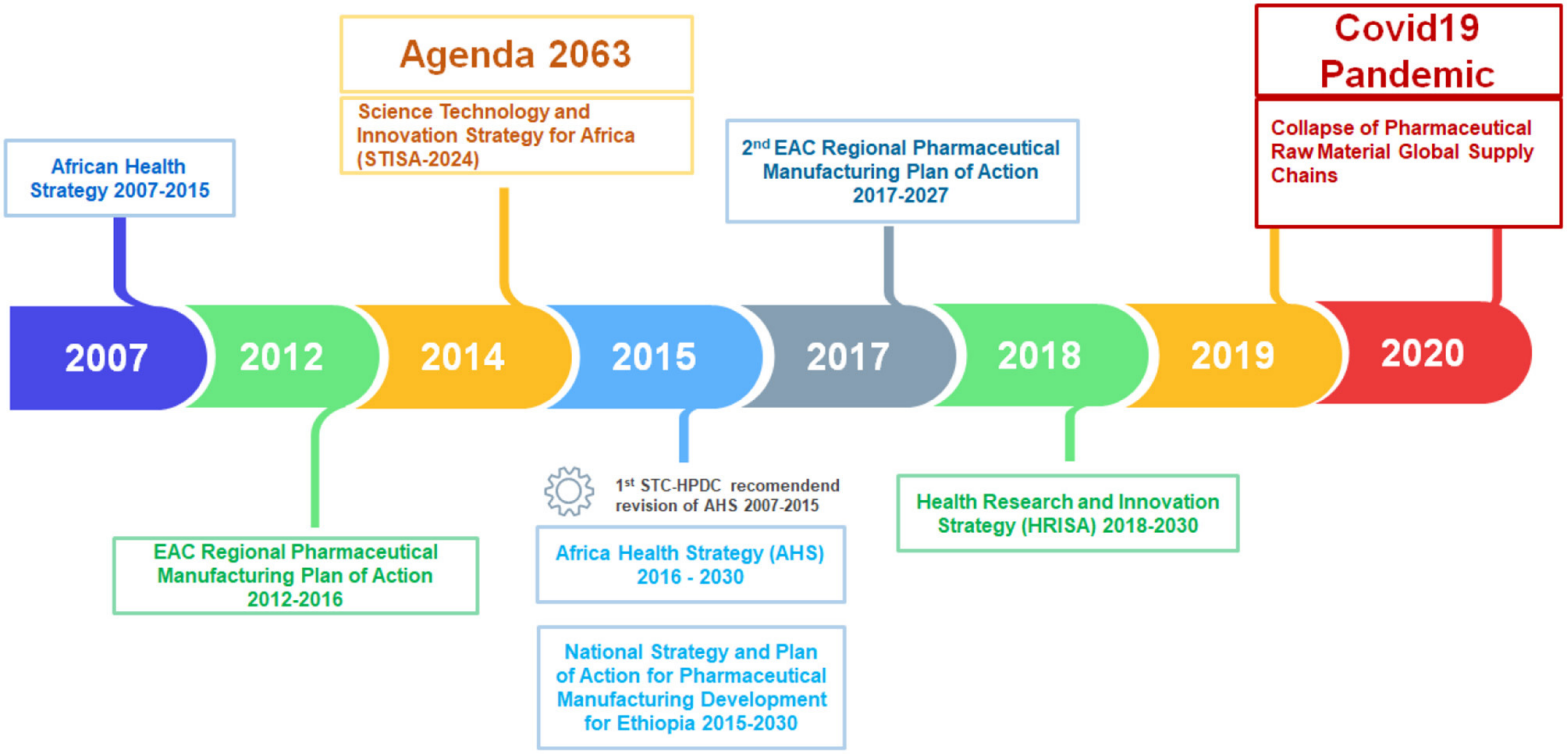
Pharmaceutical policies, initiatives, and related programs developed at the supranational level. Source: Compiled by author from various publications and interviews.

The second observation is that supranational bodies lead policy-making and strategy development for the sector (see Figure 1). A supranational organization policy-maker explained that some member countries do not have the requisite technical, institutional, and policy capabilities mix to design policy at the national level. They domesticate supranational developed policies and strategies. The AU, through its technical implementation arm AUDA/NEPAD, has built a comprehensive network of experts across the globe with deep knowledge and context of Africa. They draw on them to design supranational policies and strategies. However, countries with strong institutions and capabilities develop their pharmaceutical sectoral policies and strategies.

Local pharmaceutical production has been subdued because health systems approach view medicines as commodities that can be easily imported and not a public policy tool that can be used to support industry development (Mackintosh et al., 2018), hence the low priority given to developing policy-making skills for local production. These approaches have historical origins and were entrenched in the colonial health system—global value chains and medicines international trade traditions.

### 5.2. Local API manufacturing challenges

There is a dearth of API manufacturing on the continent, with only Aspen in South Africa producing APIs for paracetamol, codeine, and a cancer drug. An interviewee reported that even the paracetamol API production in South Africa at one point was on the verge of collapse because of cheap imports. The government had to intervene for the plant to remain viable. Evidence from our research shows that API and excipients constitute 70–90% of ex-factory costs for ARV (antiretroviral drugs), and they are all imported from India or China.

Economic, technological, environmental, business models, coordination, collaboration, and linkage capability shortcomings are reported as key impediments to local API manufacture.

**Figure 2:**
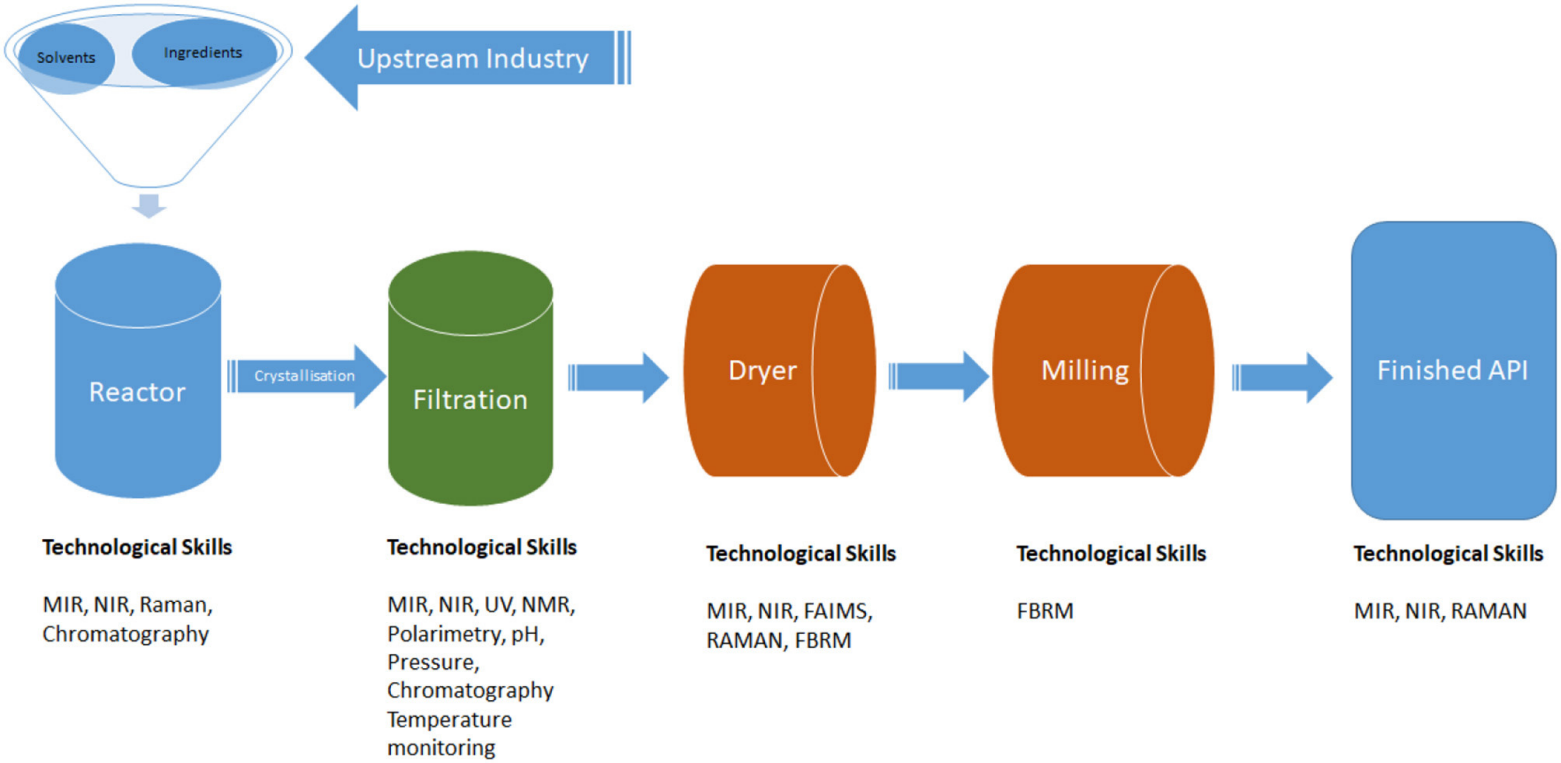
Schematic representation of the API production process and the technological skills required. Key: MIR, Magnetic Infrared Spectroscopy; NIR, Near Infrared Spectroscopy; Raman, Raman Spectroscopy; UV, Ultraviolet; NMR, Nuclear Magnetic Resonance; FAIMS, Field Asymmetric Ion Mobility Spectrometry; FBRM, Focused beam reflectance measurement. Source: adapted from Yazdanpanah (2021).

API manufacturing requires competencies in chemistry, physics, chemical, and process engineering. These specific fields are important for managing production and analytical processes. Analytical techniques used in API manufacturing include MIR, NIR, NMR, UV, FAIMS, FBRM, Raman, polarimetry, chromatography, and other temperature and pressure monitoring parameters to ascertain the fidelity of manufacturing process and quality of raw materials, intermediates, and the final products (see Figure 2).

Chemistry, physics, and engineering disciplines were not emphasized during the colonial era. For example, blacks were not allowed to study engineering and other craft areas in South Africa. If they studied elsewhere, white-owned local firms would not employ them. Thus colonialism impacted people directly by shaping institutions of knowledge generation and development while excluding blacks from technological change and critical professions (Muller, 2018). Thus, at independence, many countries had few engineers. A speaker at the Julius Nyerere Centenary Celebration at The University of Edinburgh on 7 November 2022 reported that Tanzania had one local engineer at independence. In recent years, African governments have begun promoting STEM (Science, Technology, Engineering, and Mathematics) programmes to address these key skills scarcity drivers of industrial underdevelopment.

In addition to the absence of local skilled technocrats, there were historically very low university–industry linkages in colonies (MacLeod, 1996). This persists to date. A vaccine manufacturing R&D scientist echoed and affirmed this challenge by reporting that they had not collaborated with local universities on all of their development work. They have to develop in-house R&D skills; a slower process for learning and competence building, and high direct investment in training.

Energy and environmental waste treatment costs are further challenges to local API manufacture. China overcame these challenges through the state by offering subsidized energy costs, building specific industrial parks, and seeking joint venture partners for technology transfer. The scale may be an issue, however, a respondent countered this argument and explained that a modular approach to production techniques allows a gradual build-up of scale and avoids early investment in expensive industrial infrastructure. The respondent argued that entrepreneurs need to become ambitious, break free from focusing on their country markets only, and actively target lucrative international markets. API local production requires soft and hard infrastructure investment, incentives, and frameworks that support emerging industries.

### 5.3. Financing innovation and research, industry development, and entrepreneurship

Finance is critical for stimulating and sustaining technological change-driven innovation across basic and applied research, commercialization of new technologies, and funding entrepreneurship.

#### 5.3.1. Financing innovation and research, and industry development

We reported elsewhere that many African countries are only now establishing Science Granting Councils (SGCs) and local funding is subdued (Mugwagwa and Banda, 2021). The bulk of research and innovation funding is allocated to agriculture and health, with the larger proportion externally funded (Chataway et al., 2019). Governments, except for a few such as South Africa, do not fund basic and applied research, and consequently, research priorities are set outside by the SGCs of funding countries (Chataway et al., 2019). This is not a new phenomenon. In the late colonial period, health and agriculture research institutes in east, west, and central Africa establishment were funded by the 1940 CDW (Colonial Development and Welfare). Act (Clarke, 2018).

Inaccessibility of long-term loans at the right price for upgrading facilities was a common issue raised by all respondents. Emphasizing the importance of finance, a respondent reported that existing facilities are old and expensive to retrofit for cGMP compliance. Our research over the last 12 years indicates that commercial bank-dominated financial systems are unable to fund higher pharmaceutical technology production. The financial systems architectures of especially Anglophone countries mimic those of colonial Britain where banks did not fund industrial development.

**Figure 3:**
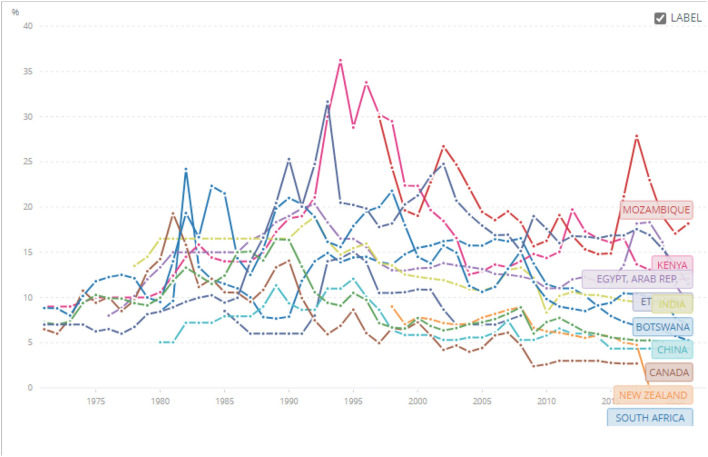
Lending rates (%) from 1971 to 2021 for Australia, Botswana, Ghana, Ethiopia, Kenya, China, Egypt, India, Mozambique, Canada, New Zealand, and South Africa. Source: World Bank Data: https://data.worldbank.org/indicator/FR.INR.LEND?end=2021&locations=BW-GH-ET-KE-CN-EG-IN-MZ-CA-NZ-ZA-AU-NG&name_desc=false&start=1971&view=chart&year=1971.

The second common issue raised by bankers and industrialists is the high cost of capital (lending rates) in Africa. We tracked lending rates for two sets of countries; those we argue had extractive institutions (African countries) and those with inclusive institutions—the neo-Europes: Canada, New Zealand, and Australia. We purposively included China and India because in the last 20 and 50 years, respectively, they built highly competitive pharmaceutical sectors (Figure 3). Figure 3 shows that since the 1970s, the lending rates in the neo-Europes have consistently been lower than for Mozambique, Kenya, Egypt, Ethiopia, and Botswana. India and China's interest rates were much better than African countries but still a little higher than Canada, New Zealand, and Australia. South Africa though is an outlier and the sanctions imposed on the country and the resultant import substitution policies drove a deepening of their financial institutions. These dynamics were and are still shaped by the lack of financial deepening and dependence on foreign capital for capex or foreign direct investment (FDI) by headquarters establishing subsidiaries or joint ventures.

In previous work, we showed that local companies incur higher interest rates compared to subsidiaries of MNCs. International banks argue that subsidiaries of MNCs have parental support to fall back on in the event of default, and from a portfolio perspective, international banks benefit from global banking relationships hence the lower interest rates. We called this the politics of lending and argued that it makes finance more expensive for local entrepreneurs (Banda, 2013b) and further locks them into industrial underdevelopment.

The third finding is the high cost of offshore funds required for the importation of plant, equipment, and machinery. The moment offshore funds cross borders into African countries they attract an “Africa Country Risk Premium” as illustrated below:

“The moment money crosses into Africa there is a country risk premium that we have to shoulder and being an African entity that raises debt on the Euro market to on-lend in Africa we still are subject to that premium. So in short therefore our rate will fall somewhere in between but inclined to the international side.” (Senior Manager, Continental Development Bank)

**Figure 4:**
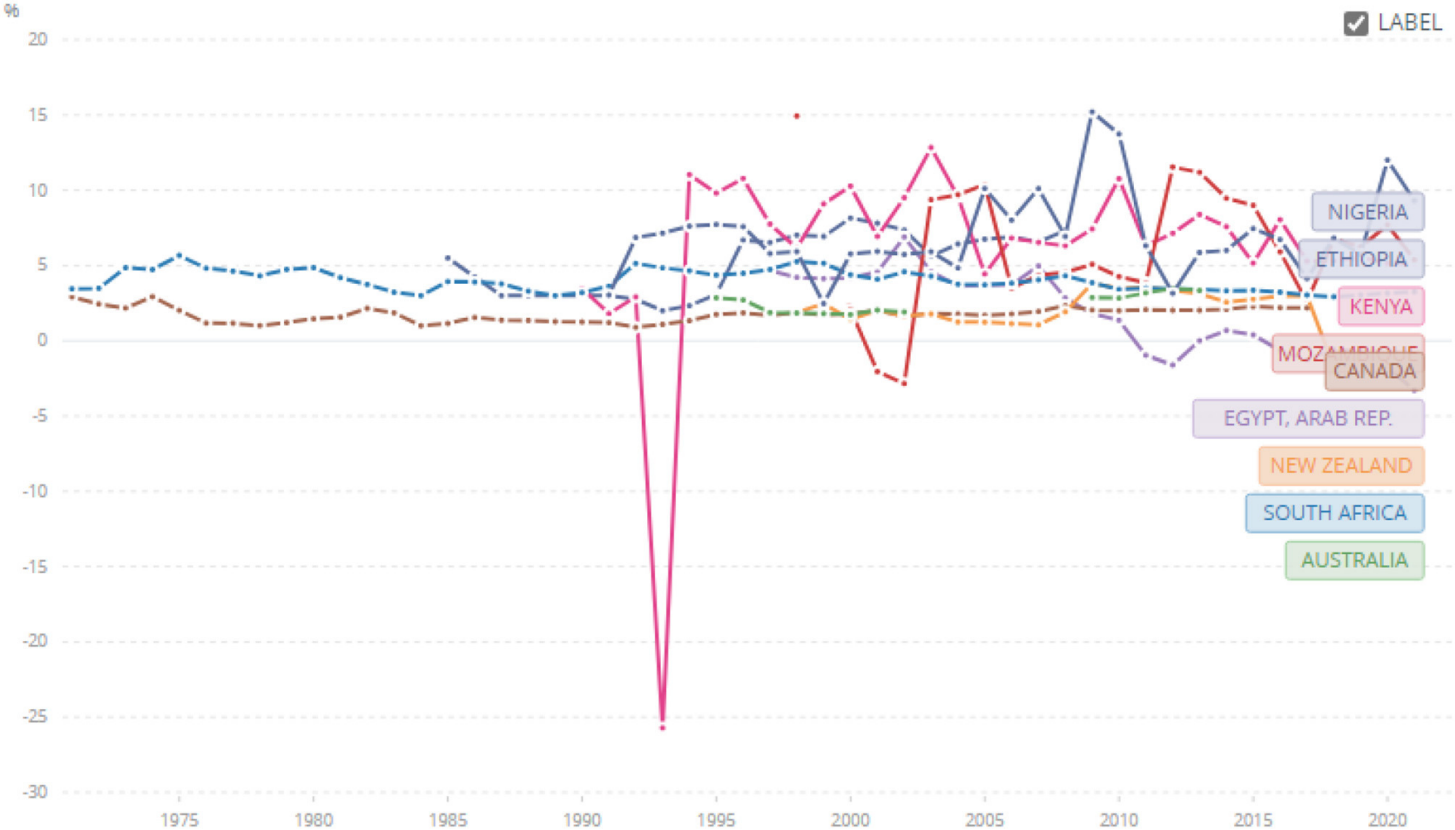
Risk premium on lending (lending rate minus Treasury Bill rate, %) from 1971 to 2021 for Australia, Botswana, Ghana, Ethiopia, Kenya, China, Egypt, India, Mozambique, Canada, New Zealand, Nigeria, and South Africa. Source: World Bank Data - https://data.worldbank.org/indicator/FR.INR.RISK?end=2021&locations=BW-GH-ET-KE-CN-EG-IN-MZ-CA-NZ-ZA-AU-NG&name_desc=false&start=1971&view=chart&year=1971.

World bank data on risk premiums calculated as lending rate minus treasury bills also shows that even for domestic debt, the risk premium in our tracer African countries is still high compared to the neo-Europes since the 1990s (Figure 4). Consequently, African industrialists are impacted by a double blow of Africa Country Risk Premium for offshore forex borrowings, and high-risk premium for domestic borrowings for working capital needs. These high financial costs drive high ex-factory costs.

To avoid expensive foreign currency loans for capex, companies resort to using internally generated profits, which take long to accrue, to fund capex needs as illustrated below.

“… to save for a USD 700K machine it can take more than a year or 2 years…. raw materials are expensive and sometimes when we get foreign orders, the money goes to buy raw materials, sometimes we have to divert that money to say salaries or something else” (Production Manager, Pharmaceutical Company)

These are manifestations of colonial financial system architectures that were set up to support commerce and trade and not industry development. Financing capex needs through small-retained earnings are suboptimal for rapid and sustained industrial development.

#### 5.3.2. Dominant local financial institutions are not designed to finance industry development and entrepreneurship

**Figure 5:**
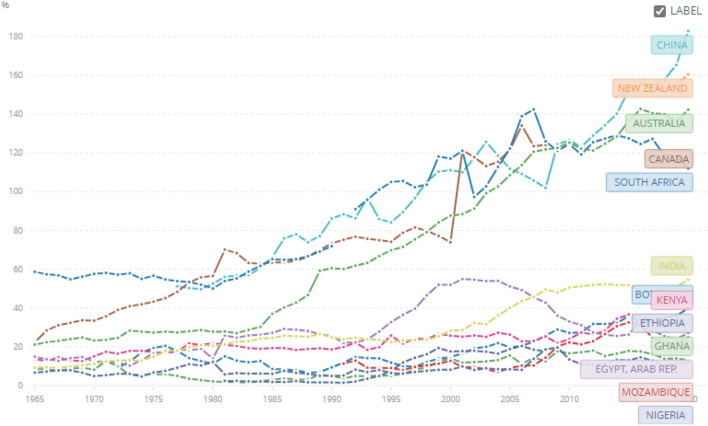
Domestic credit to the private sector (% of GDP) from 1965 to 2020 for Australia, Botswana, Ghana, Ethiopia, Kenya, China, Egypt, India, Mozambique, Nigeria, Canada, New Zealand, and South Africa. Source: World Bank Data: https://data.worldbank.org/indicator/FS.AST.PRVT.GD.ZS?end=2020&locations=BW-GH-ET-KE-CN-EG-IN-MZ-CA-NZ-ZA-AU-NG&name_desc=false&start=1965&view=chart&year=1971.

Earlier we argued that financial deepening is important for catalyzing economic development and vice versa. There is low financial deepening and the types of financial institutions dominating African countries, commercial banks, were not set up to finance industrial development. In interactions with industrialists and bankers in the west, east, and southern Africa, they all reported that medium- and long-term finance is very rare, and if available, very expensive. The most common financing instruments are short-term with a tenor of at most 1 year. Sometimes these are rolled over and become a de-facto medium or long-term financing; however, the interest rates are unsustainably high. Second, commercial banks do not avail adequate credit to local enterprises. Figure 5 shows that Neo-Europes avail much more credit as a percentage of GDP to the private sector compared to our tracer African countries. The local private sector is starved of credit (credit rationing) and this hampers industrial growth.

The origins of credit rationing are historical and are caused by shallow financial systems. Even after independence, financial deepening has not been accelerated in many countries except for a few.

### 5.4. Importance of early-stage technology support, de-risking, and value chain gap bridging institutions

**Figure 6:**
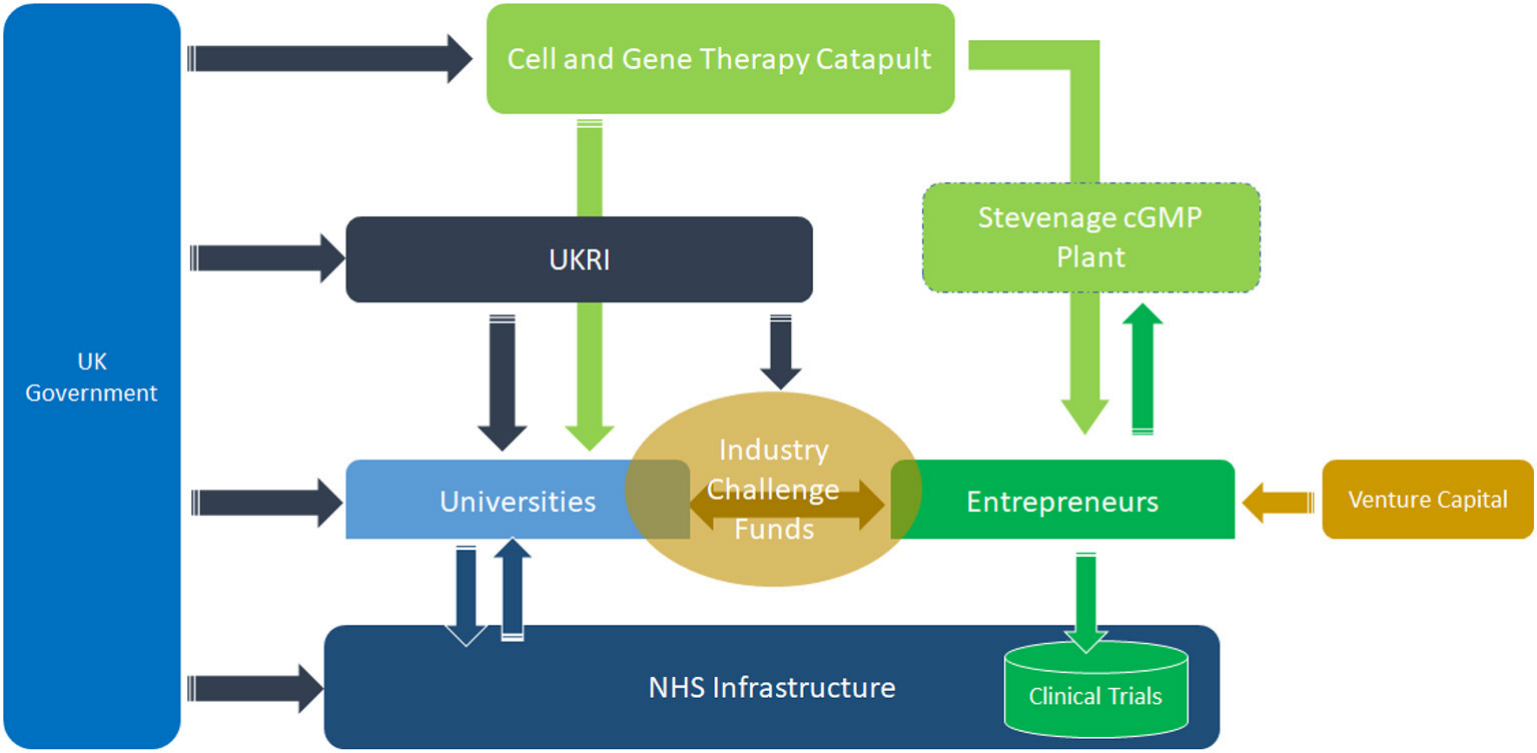
Institutions that support value chain bridging and early-stage de-risking in the UK regenerative medicine sector. Source: Author based on interviews on the RegeNableMed project.

In this section, instead of focusing on challenges, we use a positive example from the REGenableMEd project to illustrate how the identification of gaps in the regenerative medicine sector's institutional infrastructures and value chains triggered the formation of new institutions. These new institutions address technological, policy, infrastructural, and entrepreneurial challenges for the emerging innovative technology. In the REGenableMed project, we investigated the business models, value chains, and innovation ecosystems in the Regenerative Medicine sector in the UK from 2014 to 2017 (Banda et al., 2018, 2019a,b). Although not a perfect model, it is still useful in illustrating important approaches to institution building to promote the development of radical innovative technologies (Figure 6).

The government through various agencies funds the UK Research and Innovation (UKRI), the Cell and Gene Therapy Catapult (CGTC), entrepreneurs, universities, and the NHS infrastructure. These institutional interlinkages have been pivotal for the development of the UK regenerative medicine sector. UKRI also funds industry challenge funds, which facilitate university and industry collaborations. The CGTC was set up to support the sector through the provision of advice, collaborations, and seeking co-funding to develop technologies and innovations. UKRI has a budget allocated year on year that it uses to shape innovation trajectories. At the same time, universities, a key arm of the triple helix, are also funded by the government. Thus, the government funds institutions of knowledge generation, technology development, and commercialization, as well as innovation-bridging institutions such as the CGTC (Banda et al., 2018, 2019a,b). The argument is that if existing institutions have neither the capabilities nor reach to support radical innovations, then new institutions are built and purposed to support new technologies.

The CGTC through the cGMP plant in Stevenage is an innovation broker and technology early-stage de-risking agent (Figure 6). Building a cGMP is an expensive and high-risk venture for a resource-constrained entrepreneur because the technology can fail, yet there are sunk costs in the infrastructure and its maintenance costs can be as high as GBP250 000 per annum. State investment in the Stevenage plant allows entrepreneurs to manufacture small batches by renting out space and time—which is cheaper than using their premises. This de-risking function by the CGTC helps accelerate technology development and commercialization through the production of batches for clinical trials with minimal upfront investment in physical infrastructure by entrepreneurs. The entrepreneur can demonstrate proof of concept, efficacy, and safety, attributes that make their value proposition more attractive to venture capital and other early-stage funders. African pharmaceutical innovation ecosystems for drugs, vaccines, medical devices, and diagnostics lack such institutional and infrastructural support ecosystems. South Africa though stands out because of the collaborative and supportive roles played by the National Research Foundation (NRF), Council for Scientific and Industrial Research (CSIR), and Department of Trade and Industry (DTI).

### 5.5. Building local regulatory and technological capabilities

The last institutional challenge we focus on is new innovative technologies and regulatory capabilities. Table 1 shows that regulatory capabilities in the drugs sector are more developed than for vaccines, which, in turn, is higher than for medical devices. There is minimal regulatory expertise in newer technologies such as mRNA vaccines or other biologicals. The medical devices sector is hugely impacted by the absence of Notified Bodies. They rely on European Standards Bodies to be certified, although their premises are inspected by local medical regulatory agencies. At the regional level, the regulatory collaboration among Zambia, Zimbabwe, Botswana, and Namibia (ZAZIBONA) has seen skills transfer through mentoring of regulators during regulatory exercises.

**Table 1:** State of regulatory competencies for drugs, vaccines, biologics, and medical devices.

	**Number of countries with manufacturing capability**	**Number of plants**	**Regulatory competencies**
Drugs	23	653+	Yes but varying competency levels
Vaccines	4	9; RSA-−3, Tunisia-−1, Senegal-−1, Egypt-−1	Yes in specific countries
Biologics	No data, however North Africa manufactures monoclonal antibodies	No data available	Yes in North Africa and to some extent South Africa
Medical devices	No data available	No Data Available	Only facility inspection, there are no Notified Bodies on the continent and entrepreneurs depend mostly on European Standards Bodies which are Notifying Bodies

Banda et al. (2022).

Evidence suggests that even in the drugs sector, companies are burdened with multiple regulatory compliances. The recently promoted WHO pre-qualification system poses challenges to local producers. A production manager of a pharmaceutical company that had attained WHO pre-qualification reported that:

“*It's very expensive to be* WHO*-compliant to say the least, because it takes a lot of knowledge and it also takes a lot of money in terms of capital for the infrastructure*.” (Production manager, pharmaceutical company)

After an investment of over USD 2.5 million in factory upgrades, he went on to say:

“*We pay for all their [inspectors] overall upkeep, costs (flight, visa, hotel, transport) depending on where they are coming from, if they are coming from Europe it can cost about USD 50 to 60K.” (Production manager, pharmaceutical company)*

The same company bemoaned the fact that after investing in WHO pre-qualification, they went for over a year without winning a tender and the regulatory burden fell on local sales of low-cost generics to maintain it. What is strange is the emphasis on the credibility of external regulatory institutions over local ones. Local medical regulatory agencies have experience dealing with local companies and although certain conditions may not be met, one industrialist from Kenya argued that what is important is to have roadmaps for improvement. Recently, Rwanda was chosen to host the Africa Medicines Agency, perhaps this is part of the answer to building locally accepted regulatory institutions that cover drugs, vaccines, medical devices, and diagnostics without reliance on “international standards”, which in reality are local standards for companies in those jurisdictions.

## 6. Discussion

The pivotal argument of innovation systems is that technological change-driven innovation is important for building superior economic performance and competitiveness, and institutions are a vital component of the system. However, institutions are not value-neutral, they carry the social, political and economic objectives and aspirations of the agents who designed them. Colonial extractive economic and political institutions were shaped by imperialist objectives, and these institutions persist for a long time. Colonial extractive institutions forced local industry into subservience to colonial global value chains and global industrial structure labor distribution. This locked the pharmaceutical sector into perpetual underdevelopment. Unless radical economic and political institutions are introduced, nothing much will change.

Five institutional challenges (see sections 5.1–5.5) shaped by extractive institutions pose significant hurdles to the rise of an innovative African pharmaceutical sector. Innovative technologies do not arise by chance or serendipity; they are a result of purposive industrial development agenda setting, the creation of institutions and infrastructures, and resource allocation over long periods. Boettke et al. (2008) pointed out that the “underlying institutional framework of an economy influences its ability to progress”. We have shown that the underlying financial, technology, industry development, innovation brokerage, and regulatory institutional frameworks on the continent are problematic and these challenges can be traced back to colonial institutions. This is a consequence of over a century of colonial-embedded extractive economic and political institutions that persist even after independence. Literature shows that there is a long-run consequence of colonial extractive institutions on science policy, as well as economic and industrial development. Transition to innovative technology production requires radical institutional repurposing. Such endeavors are subject to contestations because of competing developmental objectives, especially in resource-limited settings. India, Bangladesh, and Sri Lanka used political and economic institutions to contend with backlash from multinationals and their governments for upsetting global pharmaceutical value chains and trade dynamics existing then (Lall and Bibile, 1978; Reich, 1994).

The theory of institutional persistence helps us understand some of the likely long-running technological, policy, innovation ecosystem, and market structures challenges. Acemoglu et al. (2001) argue that historically where suboptimal institutions were advantageous to colonizers and indeed new independent governments, those extractive institutions were perpetuated. There is a need to examine existing institutions and ascertain their fitness for pharmaceutical industrial transition to high-value, knowledge-intensive activities, cognisant of the fact that there is a cost to establishing and changing institutions. Acemoglu et al. (2002) argue that “institutional differences matter more when **new technologies** [our emphasis] that require investment from a broad cross-section of the society become available”. The cross-section of society includes smallholders, the middle class, and entrepreneurs. This is backed by Acemoglu et al. (2002) (p. 1234), institutional hypothesis which states that economic performance is linked to the organization of society, namely political, economic, and social institutions. Political institutions include governance structures, policy-making, and resource allocation. Social institutions with reference to health, pertain to attitudes and aspirations for universal health coverage, health as a right, and the use of public health policy as active industrial policy, what we call elsewhere as innovative procurement (Chataway et al., 2016). Economic institutions embody society's resource distribution goals, the purposive pursuit of certain sociotechnical imaginaries, and the promotion of particular technology and innovation futures. Carefully structured, they shape incentives and opportunities for investment, which, in turn, determines a country's chance of becoming wealthier.

Boettke et al. (2008) argue that successful institutional changes need to be linked to IEN institutions. Institutions for pharmaceutical policy design are scarce at the national level, consequently policy formulation for the pharmaceutical sector is mostly done at the regional and supranational levels because many countries lack the skills to do this. This in itself is not problematic, as it is a pragmatic way of building momentum in an area where nations may not have enough skills for policy design. However, countries that intend to be successful need to unlearn old habits, create indigenous endogenous institutions, and link them up with indigenous exotic institutions developed by supranational and regional bodies. The attraction of the supranational and regional bodies designing policies is they are likely to be accepted as “local” institutions and have political legitimacy; hence, they are likely to be institutionally sticky and adoptive of new institutions. They can also serve as institutional legacy unlearning spaces.

Sections 5.2, 5.3 and 5.4 demonstrate the need for interlinkages of STI and supporting financial and innovation broker institutions. Relevant institutions include those that link basic and applied research to the commercialization of technologies, namely government, SGCs, specific innovation broker institutions, and financial services (see section 5.4). Recent efforts to prioritize STEM and allocate resources to develop a pool of skills in science, technology engineering, and mathematics are welcome. However, this needs to be linked to the development of entrepreneurial activities in the upstream supplier industries for the pharmaceutical sector. Especially fine chemicals are required to produce raw materials for API and the biologics sector to produce starter materials for biologicals. This is an area where close collaborations between industry, universities and government (triple helix model of innovation) that supports university spinouts would be useful, and innovation brokers are critical for commercialisation of the new technologies. If the project consists of radical innovations then the rise of the sector must be synced with other bureaucratic operations, but it needs agile decision-making and adaptability in approaches. Importance of early-stage technology support, de-risking, and value chain gap bridging institutions section demonstrates these characteristics in the regenerative medicine sector in the UK.

The last institution considered in this paper that requires sustained investment is regulation and concomitant development of regulatory and governance skills in emerging technologies. The continent has relatively adequate skills in drug regulation; however, skills for vaccines and biologicals, as well as medical devices and diagnostics are comparably scarcer. These shortcomings were amplified during COVID-19 when African countries struggled to govern newly introduced medical devices, diagnostics, and vaccines. Relative to the other institutions, regulatory institutions may be the easiest to change as the colonial economic and political institutional hangover may not be as strong in this sector compared to STI, funding, and broker institutions setup. Regions such as SADC have demonstrated innovative collaborative efforts through ZAZIBONA, which has been extended to other countries. In addition, the anticipated emergence of the Africa Medicines Agency can take up the development of regulatory skills by working closely with the Africa CDC, national medicines regulatory agencies, and industry associations.

## 7. Conclusion

There is an urgent need to unlearn certain institutional traditions and break away from historically and colonially engineered economic development trajectories, extractive institutional setups, and circumscription of industrial activities away from high-value, knowledge-intensive manufacturing. Contemporary development machinery that builds on colonial relationships, values, and extractive institutions designed for the imperial era needs a clear disjuncture from these former extractive institutional arrangements. On the other hand, innovation systems theory needs to critically engage with the extractive institutional realities on the ground and incorporate decolonisation lenses in their applications of innovation systems frameworks to African countries, which were purposively locked into low-value commodity value chains. Innovation systems theory also needs to engage with colonial extractive economic and political institutions and how they shaped attendant STI infrastructures' current de-emphasis and underfunding and industrial underdevelopment for the pharmaceutical sector. The political economy of the African pharmaceutical sector is inextricably connected to the colonial political economy of trade, global industrial structure labor division and hierarchy, as well as captive value chain governance that disadvantaged African players. The underlying colonial values and ideologies have persisted perversely into contemporary times. Contemporary innovation systems' approaches to promoting the local pharmaceutical industry either ignore or omit these institutional and structural issues responsible for the sector's stunted growth.

Institutions need to be understood within the social and political contexts that gave rise to them. Given that the colonial social and political system and their attendant institutions attenuated industrial and economic development, trying to use these same institutions to become economically competitive is problematic. From an international political economy perspective, power and agency were lopsided as developing countries' economic activities were circumscribed to producing raw materials for the metropole. Path dependence matters because colonial power relations optimized increasing returns from extractive institutions, which further locked-in certain institutional arrangements that benefited the metropole at the expense of the periphery. This is pivotal because it shaped enterprise ambitions or lack thereof and locked-in traditions of doing technology industry development into a technology consumptive loop. As colonies transitioned to independent states, the same agents who used to be colonial officers became development experts and academics who trained in-coming administrators, thereby entrenching ideologies and political economy approaches to economic and industrial development. Hence, the call for decolonisation and unlearning is because most countries do not have the right institutional mixes for radical innovations and technologies to be developed locally. These constitute key institutional facets of interrogation shortcomings of innovation systems theory when applied to developing country contexts.

In conclusion, that science technology and innovation play a critical role in technological change and economic development is an accepted phenomenon nowadays. However, whether and to what extent the colonial legacies, instrumentality, and direction of extractive economic and political institutions have been decolonised is a new field of research that this study calls for. Focusing only on funding, technological capabilities, and industry development, without resolving persistent extractive institutions, will not generate a sustainable transformation of African economies, especially the pharmaceutical sector.

## Data availability statement

The original contributions presented in the study are included in the article/supplementary material, further inquiries can be directed to the corresponding author.

## Author contributions

The author confirms being the sole contributor of this work and has approved it for publication.
